# Initial production prediction for horizontal wells in tight sandstone gas reservoirs based on data-driven methods

**DOI:** 10.1038/s41598-025-14468-0

**Published:** 2025-08-04

**Authors:** Jian Sun, Jianwen Gao, Kang Tang, Long Ren, Yanjun Zhang, Zhipeng Miao, Zhe Zhang

**Affiliations:** 1 College of Petroleum Engineering, Xi’an Shiyou University, Xi’an, 710065 Shanxi China; 2 Engineering Research Center of Development and Management for Low to Ultra-Low Permeability Oil & Gas Reservoirs in West China, Ministry of Education, Xi’an, China; 3 Changqing Oilfield Company of PetroChina, Xi’an, 710018 China

**Keywords:** Production prediction, Machine learning, Tight sandstone gas reservoir, Data-Driven, Fossil fuels, Natural gas

## Abstract

Accurate prediction of the initial production in horizontal wells targeting tight sandstone gas reservoirs (IPHTSG) is critical for assessing the exploitation potential of well locations and identifying reservoir sweet spots. Traditional methods for estimating horizontal well productivity exhibit limited applicability due to reservoir heterogeneity and unfavourable petrophysical properties; therefore, this study proposes the use of machine learning for IPHTSG forecasting by systematically analysing the engineering parameters and production metrics. First, an IPHTSG database is established by categorizing and compiling the collected engineering and production parameters in addition to the classified initial production data. Second, on the basis of the IPHTSG database, prediction models for the IPHTSG are developed by employing various machine learning algorithms. The dimensionality of the input data is reduced via correlation analysis of the feature parameters, and the parameters of each prediction model are optimized using a grid search and 10-fold cross-validation. Finally, the models are applied to make predictions on a test set to validate their reliability, forming a set of methods and procedures for IPHTSG prediction. Then, this work describes a case study that was conducted on the tight gas reservoir of the H8 Member in the Sulige Southeast Field (Ordos Basin). The effective reservoir length, vertical thickness, open-flow capacity, bottom hole pressure, and amount of sand inclusion from 155 horizontal wells were selected as feature parameters, with data from 140 wells used as the training set and data from 15 wells used as the test set. Six machine learning algorithms were utilized to establish models, and the relevant calculation indicators of different models are compared. Ultimately, the XGBoost prediction model, which exhibits superior performance, is selected. This model achieves a training accuracy of 95% and a testing accuracy of 93.33%, with precision, recall, and F1-score values of 95%, 94.12%, and 93.14%, respectively, and it also has a relatively short training time. The method proposed in this paper successfully realizes IPHTSG prediction, providing a decision-making basis for formulating reasonable development plans and optimizing production parameters. This interdisciplinary methodology provides a replicable template for data-intelligent decision-making in tight gas reservoir management.

**Supplementary Information:**

The online version contains supplementary material available at 10.1038/s41598-025-14468-0.

## Introduction

With the increasing global energy demand and the gradual depletion of conventional hydrocarbon resources, unconventional tight sandstone gas reservoirs have emerged as pivotal contributors to a sustainable energy supply. However, accurate prediction of the initial production rates remains a critical challenge and has direct implications for development planning, economic evaluation, and reservoir management strategies. The complexity of the reservoir geology, the interaction between fractures, and the dynamic behaviour of fluid flow during production all contribute to the uncertainty in production forecasting. Traditional methods, such as analytical models and empirical correlations, often fail to capture the intricacies of the production process in these unconventional reservoirs, and physics-based approaches typically require precise characterization of complex fracture networks and stress-sensitive permeability variations—parameters that are inherently uncertain in heterogeneous formations^1^. Furthermore, statistical regression methods, while computationally efficient, frequently fail to capture the nonlinear interactions between geological controls and completion parameters^2^. In recent years, data-driven methods have gained increasing attention in the field of oil and gas production forecasting, and some scholars have also made certain contributions in this regard^3–5^. These methods leverage large datasets generated from various sources, including well logs, production data, and geophysical surveys, to construct predictive models without relying heavily on physical assumptions or simplifying the current assumptions regarding reservoir behaviour. Machine learning algorithms have demonstrated exceptional ability in terms of handling high-dimensional datasets and identifying complex patterns within unconventional reservoirs in the field of petroleum engineering^6–13^. Current research efforts have yet to systematically integrate static geological characteristics with dynamic production parameters while simultaneously addressing three critical aspects: (1) statistically robust sample sizes, (2) comparative evaluation of multiple machine learning algorithms, and (3) comprehensive factor analysis^14–18^. In this study, we aim to investigate the application of data-driven methods to predict the IPHTSG. By analysing a comprehensive dataset including production data, well logs, and reservoir properties, we intend to develop and validate a predictive model that can accurately forecast the initial production of horizontal wells in these unconventional reservoirs. Specifically, we explore the use of machine learning algorithms to build a predictive model based on various features extracted from the dataset. The objectives of this study are twofold: first, to develop a robust and accurate predictive model for the IPHTSG by using data-driven methods, and second, to provide insights into the key factors that influence production in these unconventional reservoirs. The results of this study have the potential to improve production forecasting, optimize well design, and enhance the economic viability of tight sandstone gas reservoir development projects. In the following sections, we present the methodology used to develop the predictive model, the dataset utilized in this study, the results of the model training and validation, and a discussion of the implications and limitations of the findings.

## Methodology and workflow

### Data Preparation and processing

To predict the IPHTSG, the theoretical or empirical formulas require specified parameters. In actual development processes, various reservoir characteristic parameters or engineering parameters can typically be obtained, and many of these parameters cannot be related to the initial production of horizontal wells via analytical equations. However, machine learning methods can be utilized to explore the relationships among them in detail. Therefore, it is imperative to acquire as many of the characteristic parameters and engineering parameters related to the reservoir encountered during horizontal drilling as possible, thus forming a dataset that is rich in parameter types and sufficient in terms of data volume. Simultaneously, the dataset must be cleaned to remove erroneous, duplicate, and missing data. Second, normalization processing should be applied to the different types of parameters within the dataset. Due to the varying orders of magnitude among different parameter types, eliminating the dimensional differences between features is necessary, as it allows the model to focus more on the intrinsic patterns of the data rather than being distracted by their dimensions. This approach enhances the model’s accuracy and generalizability. Furthermore, normalization makes the model more sensitive to variations in the input data, thereby improving its stability. Finally, correlation analysis of the data is essential, as feature selection is a crucial step in machine learning that can help identify features that are highly correlated with the target variable, which may significantly contribute to the model’s predictive performance. Moreover, features that are weakly correlated with the target variable or highly redundant with other features can be identified and potentially eliminated, simplifying the model and reducing the risk of overfitting. For the target value dataset, and based on the collected characteristic parameters, the IPHTSG can be categorized according to corresponding standards or actual field requirements.

### Selection of machine learning algorithms

After data preparation and processing, a predictive model for the IPHTSG was established. Machine learning methods can be used to interpret reservoir characteristics while drilling^19–22^, and different algorithms have different effects according to the differences in the amount of sample data and the types of feature parameters. Because the difficulty and complexity of IPHTSG prediction are far greater than those of identifying reservoir characteristics, in this work, we chose the One-Versus-Rest Support Vector Machines (OVR SVMs), One-Versus-One Support Vector Machines (OVO SVMs), Random Forest (RF), Neural Networks (NN), Extreme Gradient Boosting (XGBoost), and Categorical Boosting (CatBoost) algorithms. During the process of model parameter optimization, methods such as grid search and cross-validation are commonly employed, and based the research findings of relevant scholars^23–26^, this paper adopts the 10-fold cross-validation method. For each algorithm, all of the parameters other than the optimized parameters specified in the paper were set to their default values.

#### OVR SVMs

The SVM algorithm was initially developed for binary classification. For multicategory classification, two main methods are employed to construct SVM multiclass classifiers. The direct method modifies the objective function to solve for multiple classification planes simultaneously, but it is computationally intensive and is typically applied only to small problems. Alternatively, the indirect method combines multiple binary classifiers, such as OVR SVMs or OVO SVMs.

In OVR SVMs, each category is classified into one class during training, with the remaining samples constituting the other class. For k categories, k two-class classifiers are constructed; the i-th classifier treats the i-th category as the positive class and all other categories as the negative class. During discrimination, k classifiers produce output values fi​(x) = sgn(gi​(x)). The input is classified into the category corresponding to the classifier with the largest output, with + 1 indicating a match. However, the decision function may err, and when multiple or no outputs are + 1, the largest output determines the category. However, this method involves a challenge in that training involves only a fraction of the samples, potentially leading to notable deviations influenced by the remaining samples.

#### OVO SVMs

The SVM is based on the concept of the optimal classification surface for linearly separable data, which aims to correctly separate two sample types while maximizing their classification margin. OVO SVMs represent an extension of SVMs for multiclass classification.

When developing the IPHTSG prediction model based on OVO SVMs, two key parameters are considered: C and γ. The parameter C serves as the penalty coefficient, while γ, defined as $\:\frac{1}{{\sigma\:}^{2}}$, is a parameter in the kernel function, with σ representing the standard deviation. Following a comparative analysis, the Gaussian kernel function was selected for its high flexibility^27^.

#### RF

In the 1980 s, Breiman et al. introduced the classification tree algorithm^28^, which later evolved into RF in 2001^29^. RF enhances the bagging concept by employing CART decision trees as weak learners and selecting optimal features from a subset of sample features for tree splits, thereby improving model generalization^30^.

When establishing the IPHTSG prediction model by employing RF, two primary parameters were considered: n_estimators and max_features. n_estimators represents the number of trees in the forest, where increased values improve the performance at the cost of slower execution, while max_features denotes the maximum number of features considered per tree, with smaller subsets accelerating the variance reduction but rapidly increasing bias.

#### NN

The NN is a mathematical model that draws inspiration from the architecture and functionality of biological neural networks, constituting a nonlinear and adaptive information processing system comprising numerous interconnected nodes, and a classification neural network represents a function that emerges from the composition of multilayer nonlinear functions^31^. The activation function plays a pivotal role in this system, introducing nonlinearity that enhances the network’s capabilities.

Specifically, a neural network comprises a vast array of simple processing units that are interconnected to form a complex network structure tailored for processing and transmitting information. Neural networks possess the inherent ability to automatically learn intricate data patterns, demonstrating impressive fault tolerance and adaptability. Each neuron within the network receives input signals, processes them via a weighted summation operation followed by an activation function, and subsequently generates output signals. By fine-tuning the connection weights between neurons and selecting the appropriate activation functions, neural networks can effectively learn from and adapt to diverse tasks and datasets. In this study, the sequential module in TensorFlow was employed to construct the neural network model and the number of epochs and batch size were identified as the parameters that require optimization during the model training process.

#### XGBoost

XGBoost is a boosting algorithm that combines multiple tree models into a robust classifier. It iteratively adds trees, each learning a function to fit the residuals of the preceding prediction. During prediction, each tree outputs a score, with the sample’s predicted value being the aggregate of these scores^32^. XGBoost regulates the model complexity by adjusting the learning rate of the leaf nodes after each iteration, reducing the tree weights, and enhancing the learning space for subsequent iterations. Additionally, it supports parallelization, accelerating model computation.

When establishing the IPHTSG prediction model using XGBoost, three key parameters are preferred: n_estimators, max_depth, and learning_rate. n_estimators denotes the number of decision trees, or iterations. max_depth represents the maximum depth of each tree; a higher value increases the overfitting risk, while a lower value increases the underfitting risk. learning_rate controls the step size for the weight updates during each iteration, where a smaller value slows the training speed.

#### CatBoost

CatBoost is a gradient boosting-based decision tree algorithm that is adept at handling datasets with numerous categorical features. It operates within the gradient boosting paradigm, iteratively constructing decision trees to progressively reduce prediction errors. Each subsequent tree is trained on the residuals of the prior trees’ predictions, aiming to rectify errors from earlier models. CatBoost distinguishes itself through its unique handling of categorical features, obviating the need for complex preprocessing, such as one-hot encoding.

This algorithm employs “target statistics” to manage categorical features, computing the category distributions and then utilizing those distributions as numerical features. Furthermore, CatBoost introduces ordered boosting to mitigate prediction shifts. Unlike traditional gradient boosting, which uses the same feature set for all samples, CatBoost randomly orders samples and constructs each tree based on only the feature values from the preceding samples in order. This approach enhances generalization by preventing the model from accessing future data during training.

### Construction of model libraries for predicting the IPHTSG

In the process of applying machine learning, the model performance varies with different problems and datasets, and there is no single model that exhibits optimal performance across all data scenarios. Therefore, based on the aforementioned six machine learning algorithms, we optimized the model parameters for actual data, constructed a prediction model library for the initial productivity of horizontal wells in tight sandstone gas reservoirs, and applied these models to predict the test set. By comparing the prediction results for the training set and the test set, we then selected the optimal prediction model.

### Workflow

**Fig. 1:**
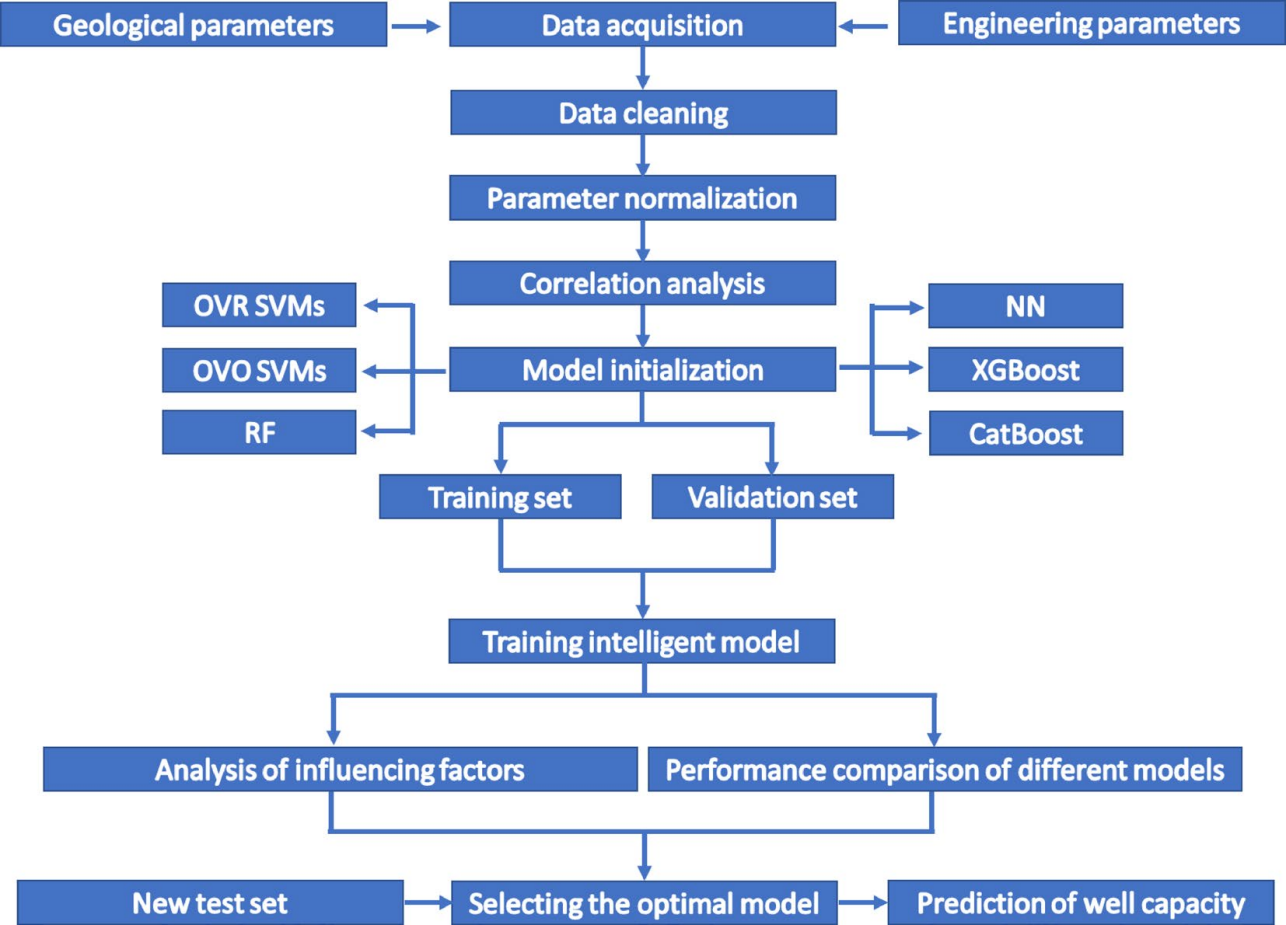
workflow.

The data-driven workflow for predicting the IPHTSG is illustrated in Fig. 1.

1. Collect information on the geological and engineering parameters for each horizontal well;
2. Perform data cleaning, normalization, and correlation analysis on the collected raw data to improve the data quality;
3. Initialize the IPHTSG prediction model with six algorithms: the OVR SVMs, OVO SVMs, RF, NN, XGBoost and CatBoost;
4. Train the IPHTSG prediction model on the training set and validation set data, optimize the parameters of each model, and compare the training performance of each model;
5. Select the optimal model for IPHTSG prediction on the basis of the test set data.

## Case study

**Fig. 2:**
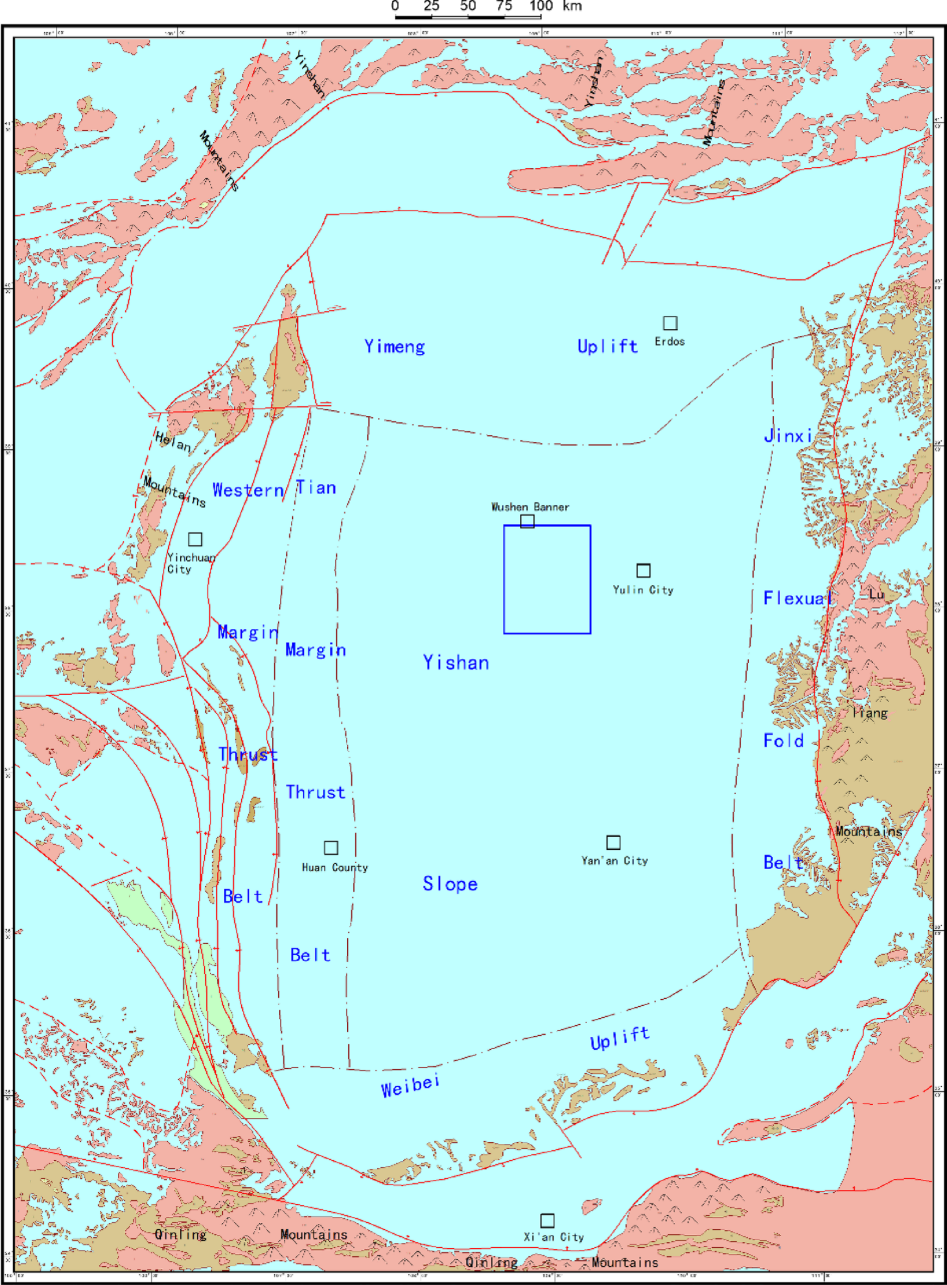
Geographical location map of the research area (created by Geomap 3.6 software).

In this study, the He-8 Member of the Shihezi Formation in the southeastern Sulige area of the Ordos Basin was selected as the research area (Fig. 2), with horizontal wells within this region serving as the research subjects. The gas reservoirs in the He-8 Member are characterized by widespread distribution and extensive gas-bearing areas. Against this geological background, the study area has established itself as the largest demonstration base for the development of tight gas horizontal wells in China. However, the He-8 Member reservoir presents several development challenges, including tight reservoir properties, strong heterogeneity, low pressure, and low single-well production rates.

Influenced by the sediment supply from the Yishan Mountains to the north of the basin, the He-8 Member in the northern part of the study area experienced a steep topographic slope during its depositional period, coupled with a gentle depositional basement and a rapid, shallow water depositional environment. This setting primarily fostered the development of a braided river depositional system with frequent lateral shifts, and from north to south, the depositional environment successively transitions from alluvial plain facies through delta plain facies to delta front facies. The primary depositional environment in the study area is the delta plain facies depositional system. The braided channel sand bodies exhibit a broad, repetitive, and extensive north‒south distribution characterized by a high sand‒shale ratio and significant sand body thickness, and the vertically stacked thickness of the sand bodies in the He-8 Member ranges from 10 to 30 m. The developmental scale and geometric morphology of the sand bodies are controlled by the width of the river channels, with variations observed in the internal sand body structures, and the primary storage spaces in the He-8 Member reservoir are dissolved pores, intercrystalline pores, residual intergranular pores, and minor microfractures. Core analysis indicates that the porosity of the He-8 Member reservoir is mainly distributed between 4% and 12%, while the permeability is predominantly within the range of 0.1 mD to 0.4 mD.

### Establishment of a database for predicting the IPHTSG in the study area

Data were collected on the relevant engineering parameters, reservoir characteristics encountered during drilling, and actual initial production capacities for 183 horizontal wells in the tight sandstone gas reservoirs of the He-8 Member, and statistical analysis of the data revealed that the reservoir horizons of these 183 horizontal wells are similar in position and physical properties. Therefore, by using only the average porosity and average permeability of the reservoirs as characteristic parameters has a limited impact in terms of predicting the initial production capacity of horizontal wells. For the engineering and production data related to the reservoirs contained in the horizontal wells, the following parameters were collected: the effective reservoir length (ERL), effective reservoir drilling rate (ERDR), vertical thickness (VT), open-flow capacity (OFC), bottom hole pressure (BHP), amount of fluid entering the ground (AFEG), and amount of sand inclusion (ASI). The ranges of these seven characteristic parameters are presented in Table 1.

**Table 1:** The range of feature parameters.

features	ERL (m)	ERDR (%)	VT (m)	OFC (m^3^/d)	BHP (MPa)	AFEG (m^3^)	ASI(m^3^)
minimum value	23	6.7647	0.16	1.42	7.745	1103.7	91.6
maximum value	2014	100	40.3	204.1815	23.6926	12415.2	1091.6

**Fig. 3:**
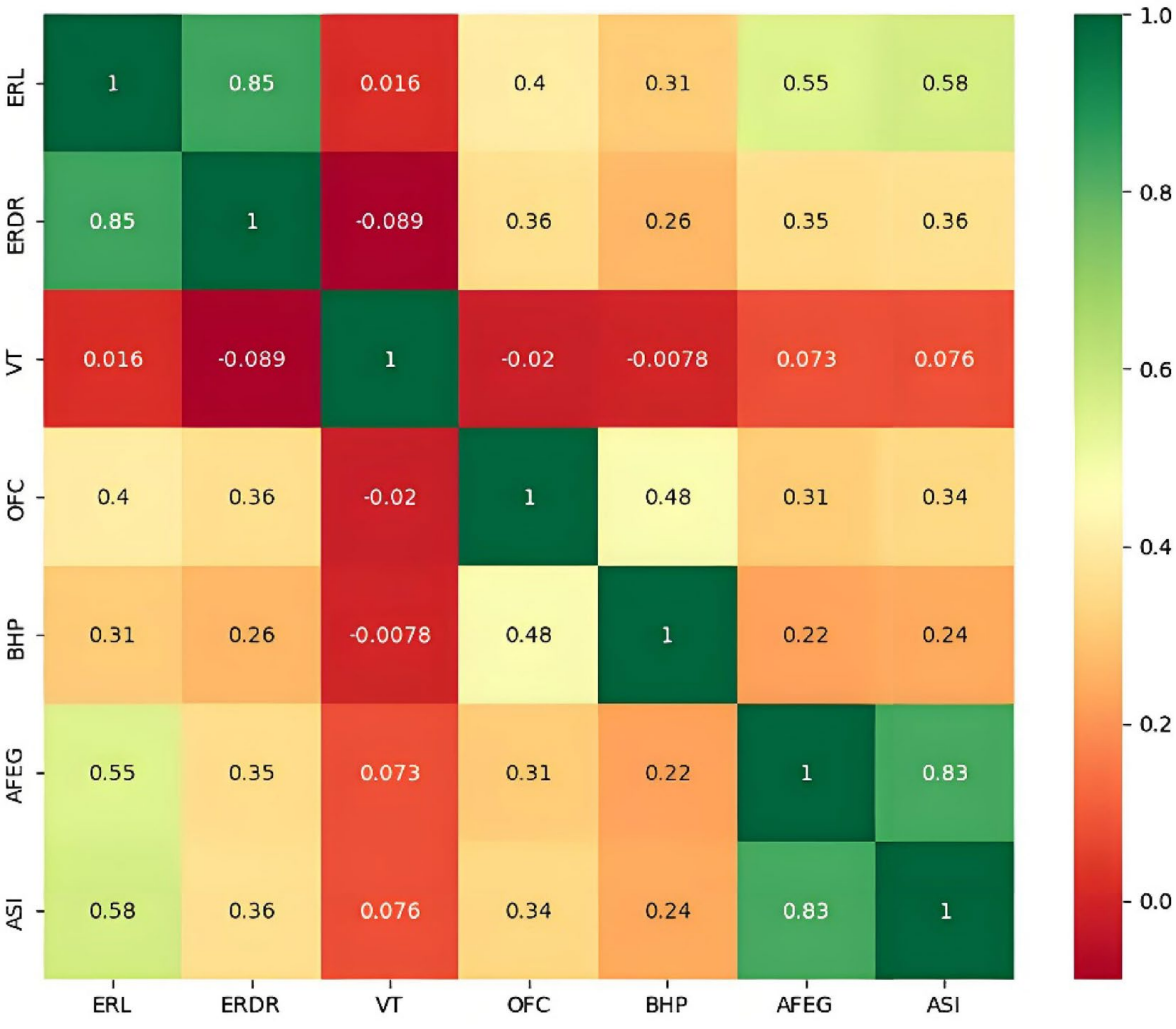
Correlation analysis of 7 kinds of logging data.

After utilizing a data analysis and processing module written in Python to clean the obtained data, a total of 155 sets of valid data were ultimately selected. The feature parameters were then analysed for correlation, as illustrated in Fig. 3, which shows that the ERL-ERDR and AFEG-ASI exhibit strong correlations, exceeding 0.8. Therefore, the ERL, VT, OFC, BHP, and ASI were selected as the feature parameters for model training.

The feature parameters were subjected to standardization processing using the fit_transform method provided by the scikit-learn library. The processing formula is $\:z=\frac{\text{x}-{\upmu\:}}{{\upsigma\:}}$, where x represents the original data, µ is the mean, σ is the standard deviation, and z represents the standardized data. The initial production rates corresponding to the 154 sets of horizontal well feature parameters range from 0.2035 × 104 m³/d to 11.8233 × 104 m³/d. These rates were categorized into six types of target values based on the following intervals: less than 2 × 104 m³/d (I), 2 × 104 m³/d to 4 × 104 m³/d (II), 4 × 104 m³/d to 6 × 104 m³/d (III), 6 × 104 m³/d to 8 × 104 m³/d (IV), 8 × 104 m³/d to 10 × 104 m³/d (V), and greater than 10 × 104 m³/d (VI). Among them, 16 wells were categorized as Type I, 42 as Type II, 50 as Type III, 32 as Type IV, 13 as Type V, and 2 as Type VI. Partial data from the horizontal well initial production capacity database are presented in Table 2. A total of 140 sets of data were randomly selected as the training set, and 15 sets were selected as the test set.

**Table 2:** Partial data of IPHTSG database.

ERL	VT	OFC	BHP	ASI	Type
−0.080711636	0.35192887	−0.738854964	−0.988969036	−0.803378161	II
0.278612158	−0.7008323	2.128916229	0.265916439	−0.492144235	V
−0.910326866	−0.696990106	−0.489416027	−0.329953277	−0.651948201	III
−1.877330606	−0.064949184	−1.204198013	−1.404648903	−1.178812808	I
−1.549711852	−0.39729897	−0.962222462	0.259283451	−1.284185729	II
−0.300004834	−0.362719223	−0.470279351	0.902417943	−0.282096226	III
−1.351555348	−0.301244119	−0.654111885	−0.071986908	−1.513075253	I
−0.619697327	−0.698911203	−0.987362	−0.570787586	0.304782097	I
−0.477024644	−0.180215006	−0.41268948	−0.049624264	−0.280700558	III
−0.691033668	1.325925062	−0.756443922	0.229870888	−0.416080338	II

### Establishment of the study area IPHTSG prediction model

After the IPHTSG prediction database for the study area was established, the selected machine learning algorithms were employed to develop the IPHTSG prediction models for the study area.

#### IPHTSG prediction model based on the OVR SVMs algorithm

**Fig. 4:**
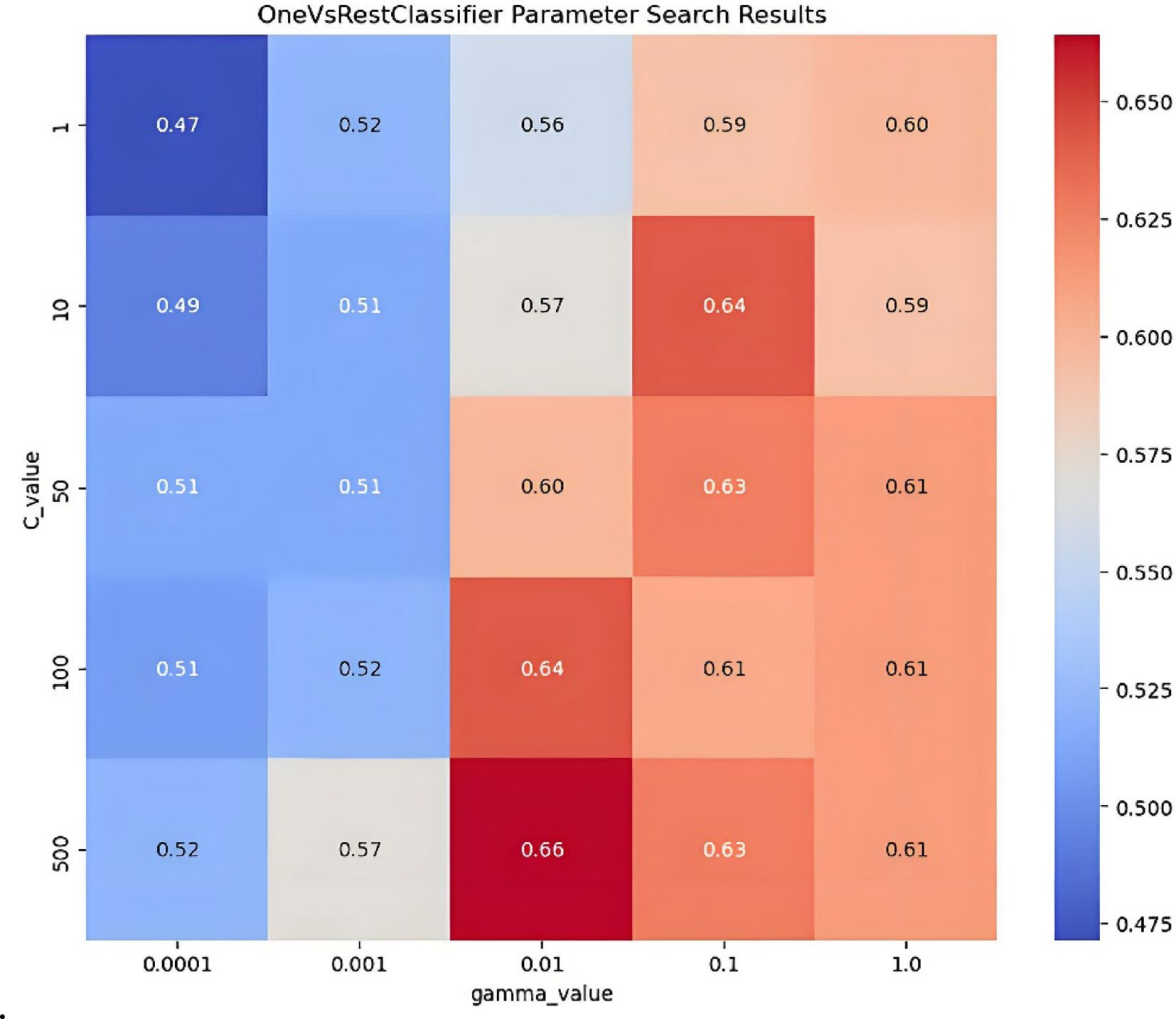
The parameter optimization process in OVR SVMs prediction model.

 The Gaussian kernel function was used to establish the OVR SVMs prediction model, and the main parameters C and γ were optimized. The values C = [1, 10, 50, 100, 500], γ= [0.0001, 0.001, 0.01, 0.1, 1] were used to search for the optimal parameter values in 25 combinations of (C, γ) using the grid search method and 10-fold cross-validation method. The parameter optimization process is shown in Fig. 4, with the highest prediction accuracy on the training set being 66%, corresponding to the optimal parameter combination of (500, 0.01).

#### IPHTSG prediction model based on the OVO SVMs algorithm

**Fig. 5:**
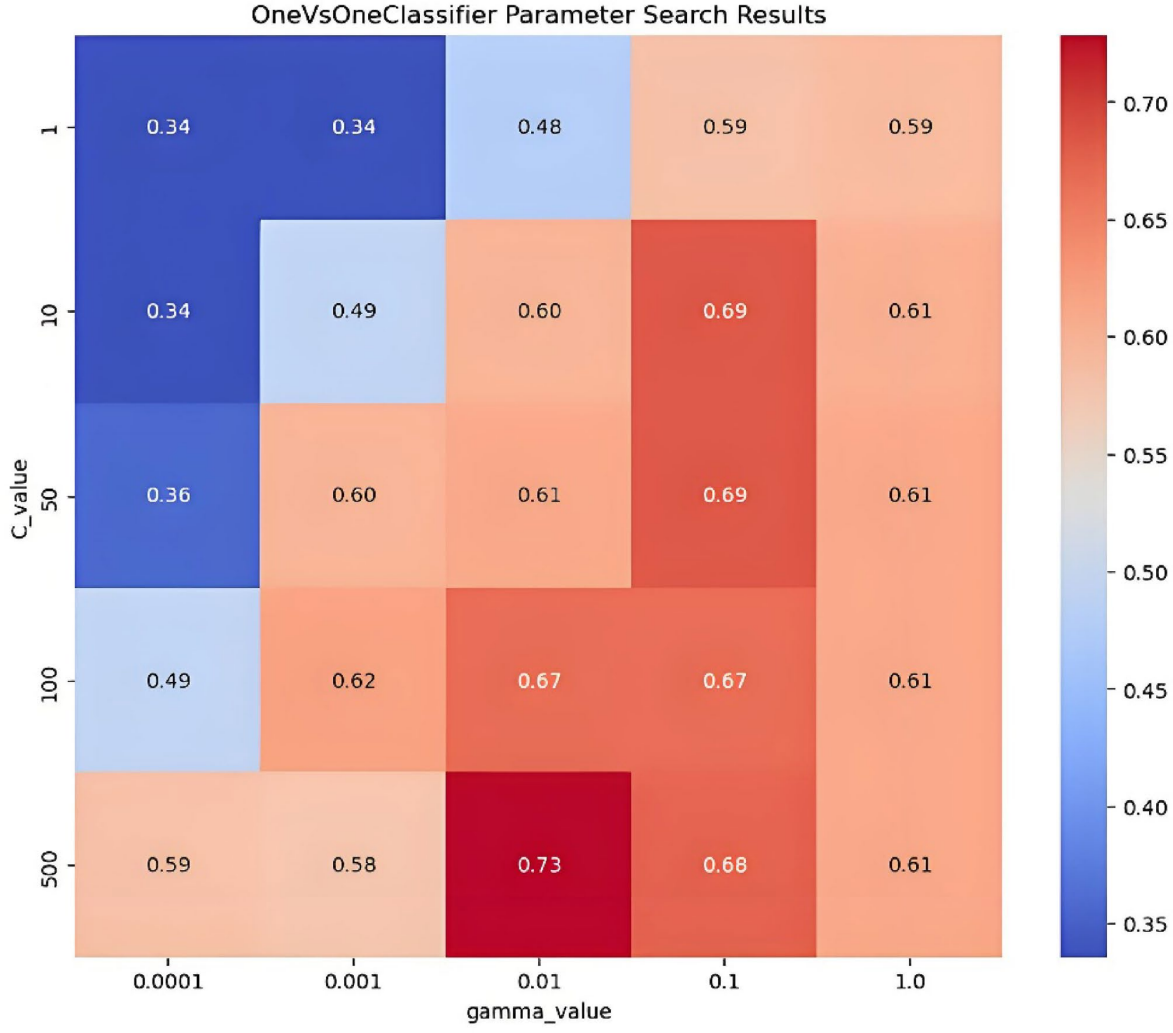
The parameter optimization process in OVO SVMs prediction model.

The Gaussian kernel function was used to establish the OVO SVMs prediction model, and the main parameters C and γ were optimized. The values C = [1, 10, 50, 100, 500], γ= [0.0001, 0.001, 0.01, 0.1, 1] were used to search for the optimal parameter values in 25 combinations of (C, γ) using the grid search method and 10-fold cross-validation method. The parameter optimization process is shown in Fig. 5, with the highest prediction accuracy on the training set being 73%, corresponding to the optimal parameter combination of (500, 0.01).

#### IPHTSG prediction model based on the RF algorithm

**Fig. 6:**
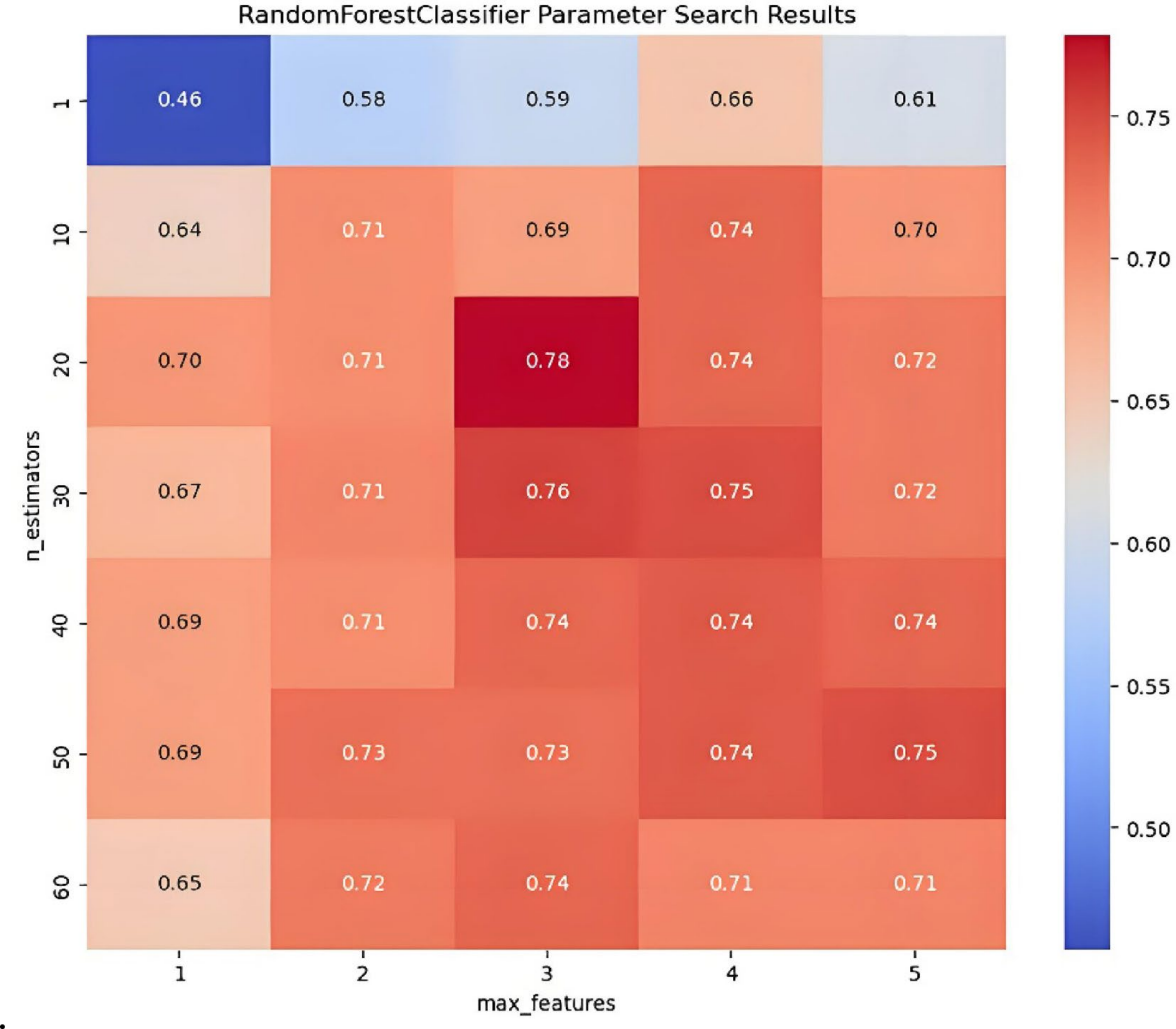
The parameter optimization process in RF prediction model.

 The main parameters n_estimators and max_features were optimized in the RF algorithm. The values n_estimators = [1, 10, 20, 30, 40, 50, 60], max_features = [1, 2, 3, 4, 5] were set to search for the optimal parameter values in 35 combinations of (n_estimators, max_features) using the grid search method and 10-fold cross-validation method. The parameter optimization process is shown in Fig. 6, with the highest prediction accuracy on the training set being 78%, corresponding to the optimal parameter combination of (20, 3).

#### IPHTSG prediction model based on the NN algorithm

**Fig. 7:**
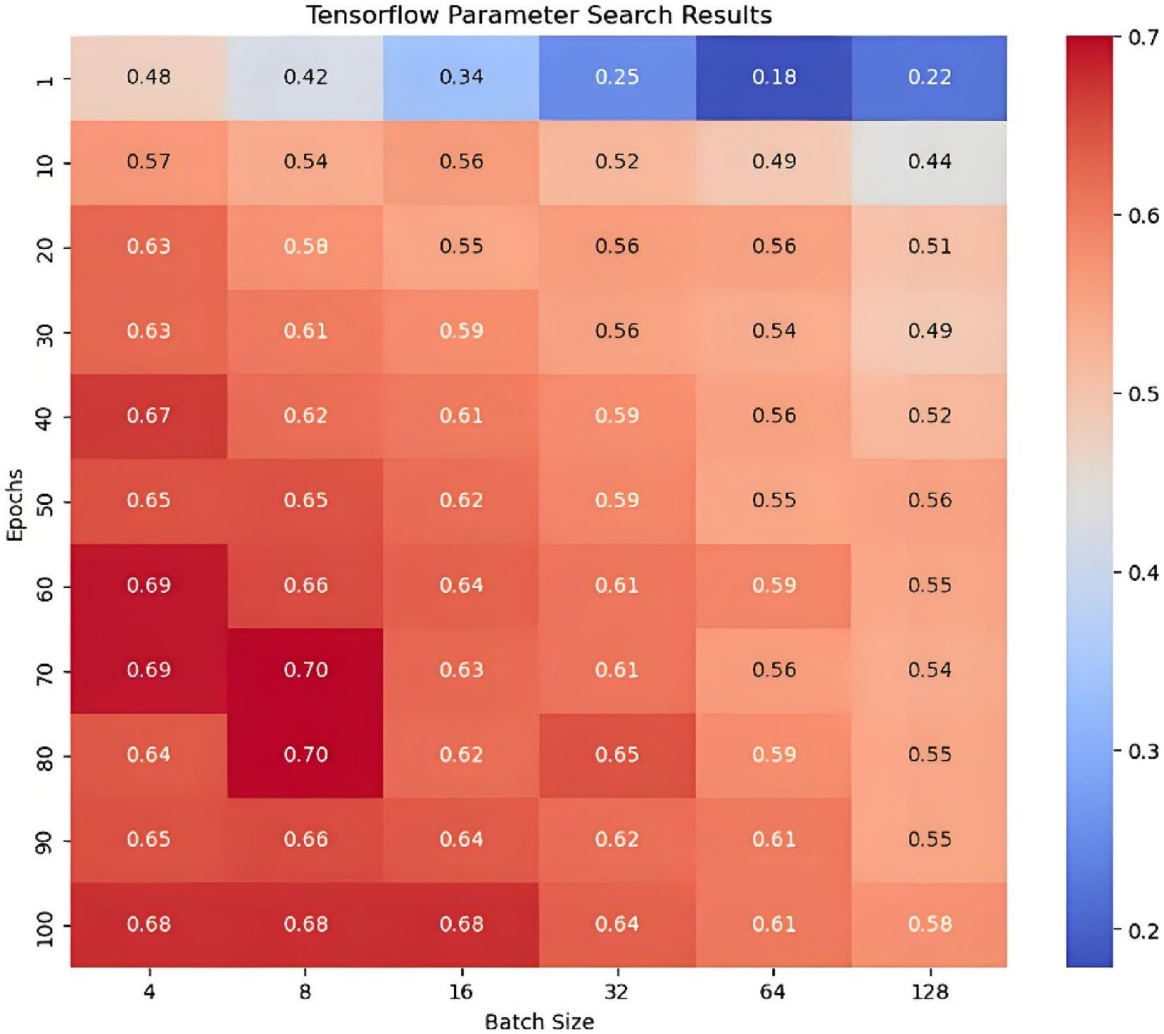
The parameter optimization process in sequential prediction model.

The sequential TensorFlow method was used to construct the IPHTSG prediction model for the study area. The main parameters, the number of epochs and batch_size, were optimized. The values epochs = [1, 10, 20, 30, 40, 50, 60, 70, 80, 90, 100], batch_size = [4, 8, 16, 32, 64, 128] were used to search for the optimal parameter values in 66 combinations (epochs, batch_size) using the grid search method and 10-fold cross-validation method. The parameter optimization process is shown in Fig. 7, with the highest prediction accuracy on the training set being 70%, corresponding to the optimal parameter combination of (70, 8).

#### IPHTSG prediction model based on the XGBoost algorithm

**Fig. 8:**
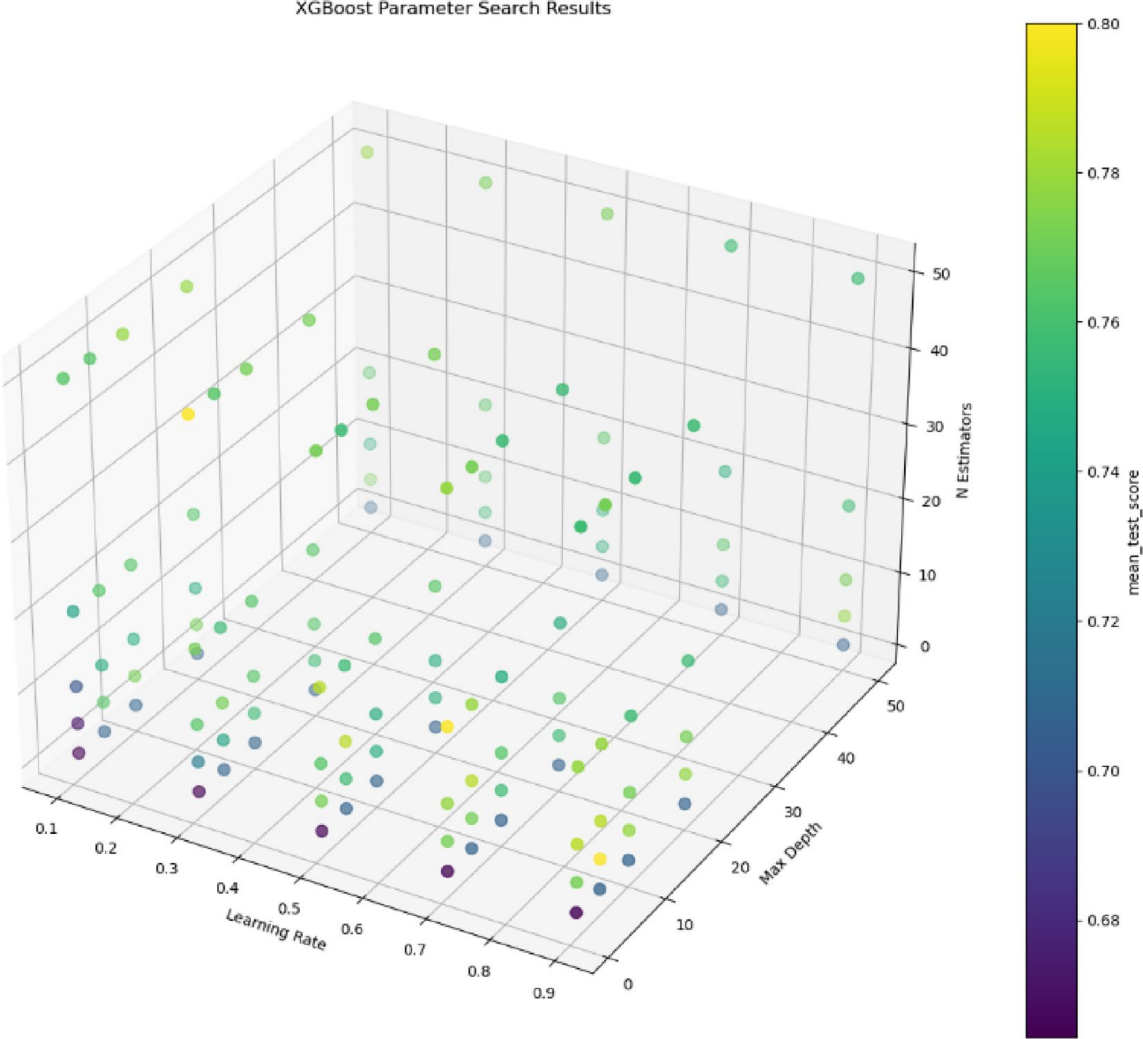
The parameter optimization process in XGBoost prediction model.

During the establishment of the XGBoost prediction model, three main parameters needed to be optimized. The values n_estimators = [1, 5, 10, 20, 50], max_depth = [1, 5, 10, 20, 50], and learning_rate = [0.1, 0.3, 0.5, 0.7, 0.9] were set to search for the optimal parameter values in 125 combinations (n_estimators, max_depth, and learning_rate) using the grid search method and 10-fold cross-validation method. The parameter optimization process is shown in Fig. 8, with the highest prediction accuracy on the training set being 80%, corresponding to the optimal parameter combination of (50, 1, 0.3).

#### IPHTSG prediction model based on the catboost algorithm

**Fig. 9:**
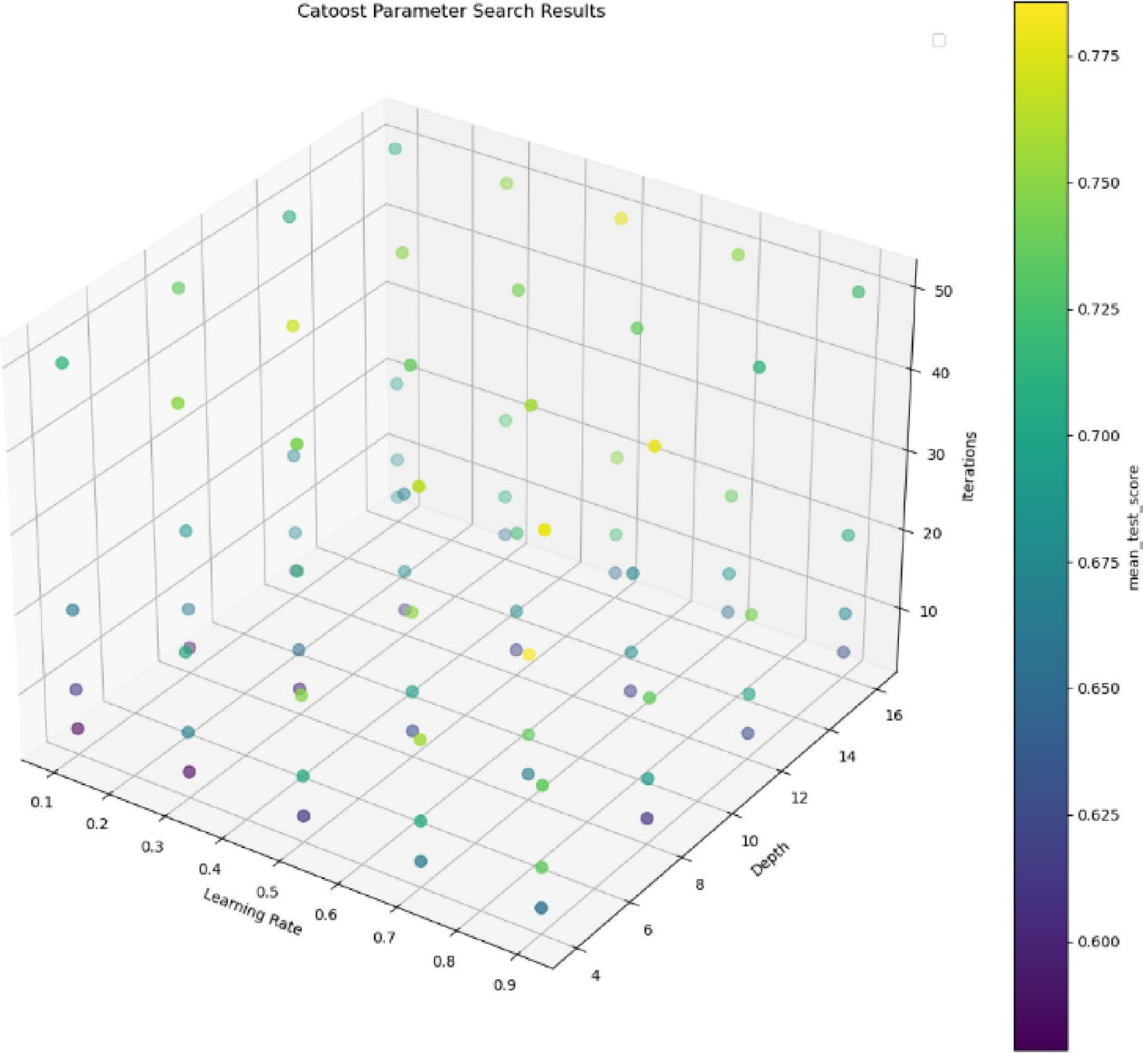
The parameter optimization process in CatBoost prediction model.

During the establishment of the CatBoost prediction model, three main parameters were optimized. The values iterations = [5, 10, 20, 50], depth = [4, 8, 12, 16], and learning_rate = [0.1, 0.3, 0.5, 0.7, 0.9] were used to search for the optimal parameter values in 80 combinations of (iterations, depth, and learning_rate) using the grid search method and 10-fold cross-validation method. The parameter optimization process is shown in Fig. 9, with the highest prediction accuracy on the training set being 78%, corresponding to the optimal parameter combination of (20, 8, 0.7).

### Comparison of the prediction results for each prediction model

**Fig. 10:**
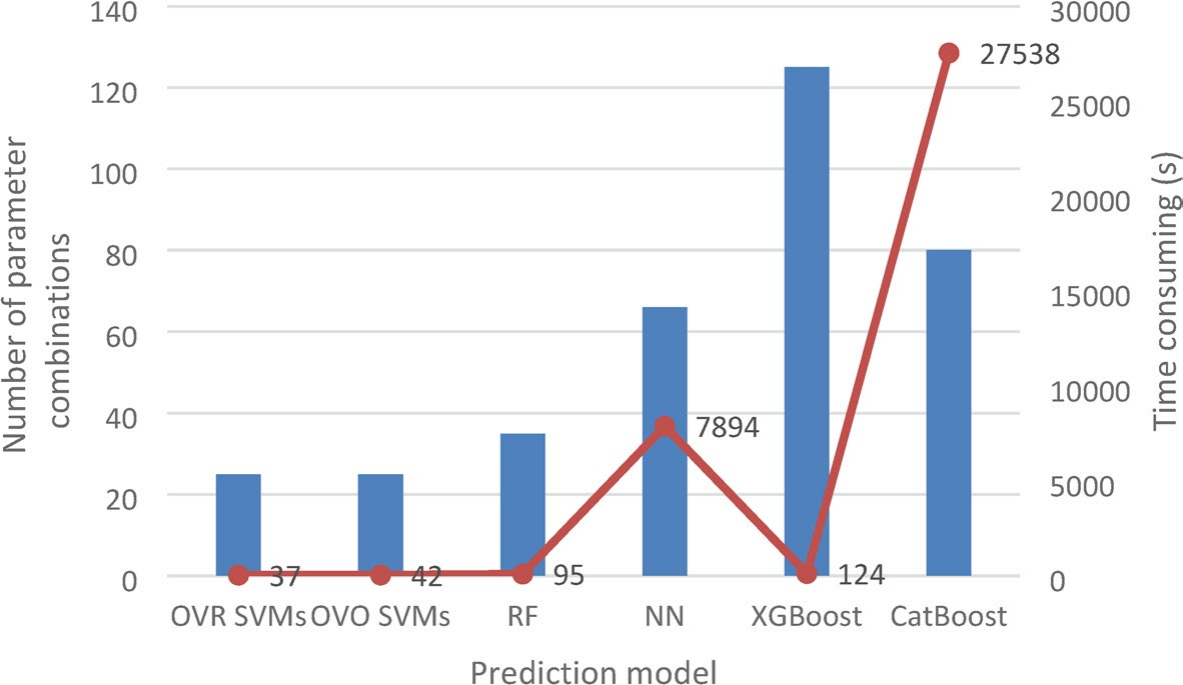
Training time of 6 prediction models.

After establishing the model and optimizing the parameters of the OVR SVMs, OVO SVMs, RF, NN, XGBoost, and CatBoost algorithms for the IPHTSG in the study area, the six models were utilized to make predictions on the test set data. The time consumed during parameter optimization for the six models is illustrated in Fig. 10, and the optimal parameters are presented in Table 3. The training set accuracy, test set accuracy, precision, recall, and F1 score for the six models are shown in Table 4.

The machine learning models in this case study were trained on hardware equipped with an i7-13700 K 3.40 GHz processor, 32.0 GB of RAM, and a 64-bit Windows operating system. As illustrated in Fig. 10, although the optimal combination of parameters for the prediction models based on the six algorithms is similar, the training time of the models varies significantly. The training time for the NN model is 7894 s and that for the XGBoost model is 27538s. These two models exhibit significantly longer training times than the other models do. Therefore, unless the performance of the NN and XGBoost models is substantially superior to that of the other models, they were preferably not selected in this case study.

**Table 3:** Optimal parameters for different models.

prediction model	optimal parameters								
	C	γ	n_estimators	max_features	epochs	batch_size	max_depth	learning_rate	iterations
OVR SVMs	500	0.01	/	/	/	/	/	/	/
OVO SVMs	500	0.01	/	/	/	/	/	/	/
RF	/	/	20	3	/	/	/	/	/
NN	/	/	/	/	70	8	/	/	/
XGBoost	/	/	50	/	/	/	1	0.3	/
CatBoost	/	/	/	/	/	/	8	0.7	20

**Table 4:** Performance of different models.

prediction model	Training set	Test set			
	Accuracy	Accuracy	Precision	recall	f1
OVR SVMs	0.8286	0.5333	0.6733	0.5642	0.5576
OVO SVMs	0.8714	0.8	0.8533	0.7999	0.8033
RF	0.9929	0.8667	0.92	0.8673	0.87
NN	0.9927	0.6	0.6857	0.6012	0.58
XGBoost	0.95	0.9333	0.95	0.9412	0.9314
CatBoost	0.9989	0.9333	0.95	0.9412	0.9314

**Fig. 11:**
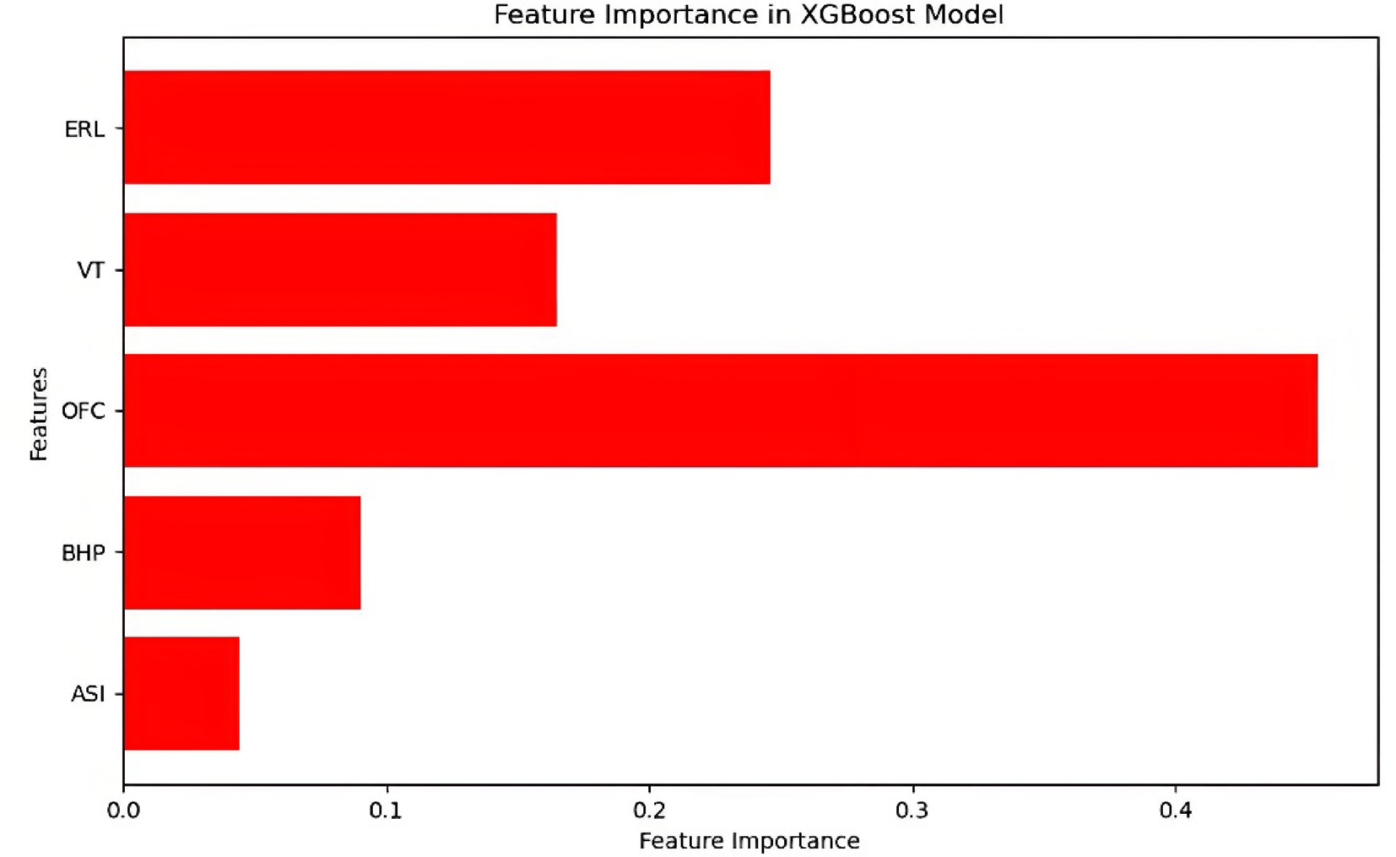
Feature importance analysis of XGBoost prediction model.

An analysis of the performance of the six prediction models was conducted, and a comparison of the accuracies on the training set revealed that all of the models, except for the OVR SVMs prediction model, achieved accuracies exceeding 0.85. Specifically, the RF, NN and CatBoost prediction models attained training set accuracies above 0.99, with CatBoost reaching the highest accuracy at 0.9989. When the accuracy, precision, recall, and F1-score on the test set are compared, it is evident that these four performance metrics are positively correlated for all prediction models. The XGBoost and CatBoost prediction models exhibited identical values for all four performance metrics on the test set, and the ranking of the six prediction models on the test set, from best to worst, is as follows: CatBoost (XGBoost), RF, OVO SVMs, NN, and OVR SVMs. Both the NN and OVR SVMs prediction models demonstrated performance metrics below 0.8 on the test set. Furthermore, a comprehensive comparison of the performance metrics calculated for the training and test sets across all of the prediction models reveals that the NN and OVR SVMs models significantly overfitted the training data, while the OVO SVMs model exhibited relatively stable overall performance but was not optimal. The RF model achieved a high accuracy on the training set; however, when applied to the test set, only the precision exceeded 0.9, with all other performance metrics falling below 0.9, indicating overfitting. Only the XGBoost and CatBoost prediction models consistently displayed high (above 0.9) and relatively stable performance metrics. Considering that the training time of the CatBoost model significantly exceeds that of the XGBoost model, the XGBoost prediction model was selected for initial production prediction in horizontal wells in this case study. We conducted a feature importance analysis of the XGBoost prediction model, and the results are presented in Fig. 11. The degree of influence of each feature on the prediction results, from greatest to least, is as follows: the OFC, ERL, VT, BHP, and ASI.

**Fig. 12:**
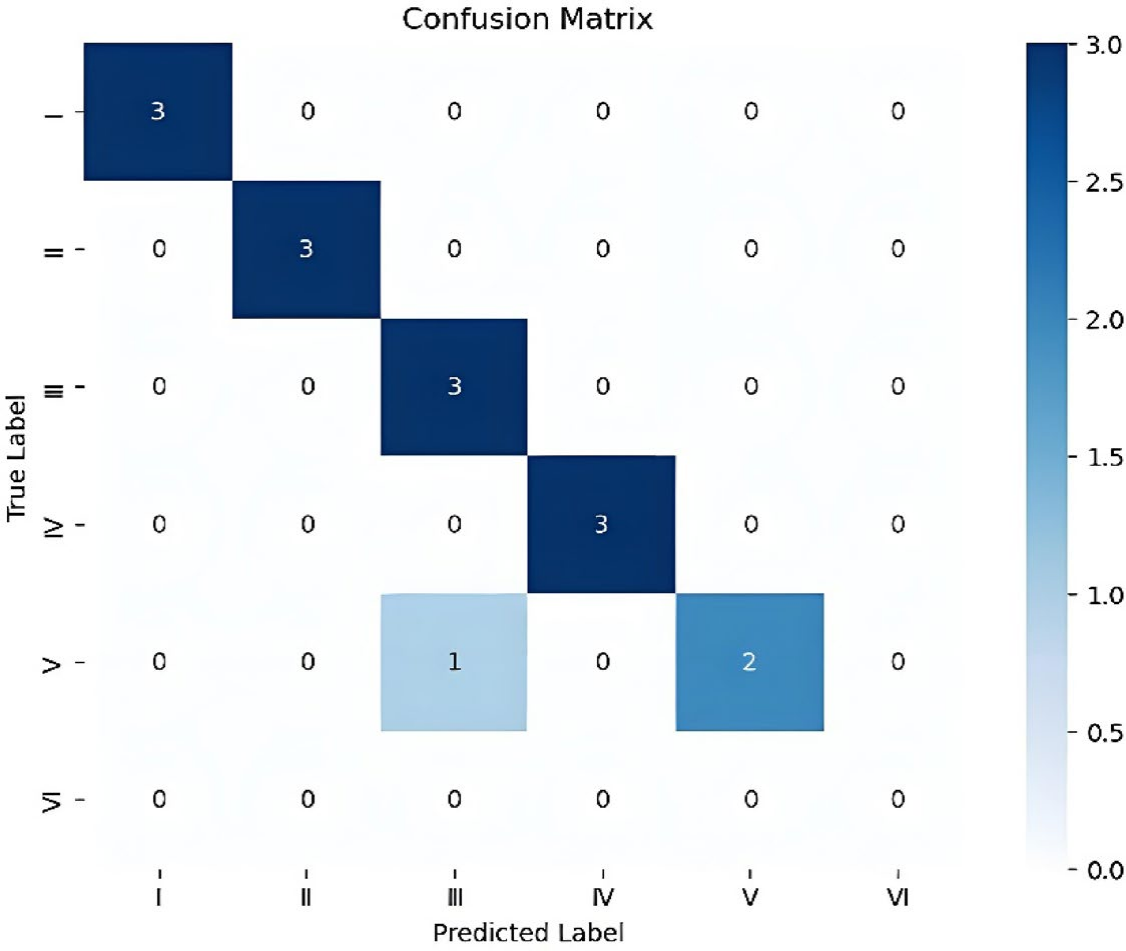
Confusion matrix for the test set of XGBoost prediction model.

The confusion matrix (Fig. 12) and Prediction performance for each category (Table 5) for the test set predictions made by the XGBoost prediction model reveal that the model achieves 100% accuracy when predicting horizontal wells with an initial production rate category of I, II, III and IV. For horizontal wells with an initial production rate category of V, the prediction accuracy is 66.7%. For Categories I, II and IV, the precision, recall, and F1-score are all 1. For Category III, the precision is 0.75, the recall is 1, and the F1-score is 0.8571. For Category V, the precision is 1, the recall is 0.6667, and the F1-score is 0.8. Due to the limited amount of data, there is no data for Category VI in the test set. These results indicate that the XGBoost prediction model can accurately predict horizontal wells with an initial production rate less than 8 × 10^4^m³/d in the study area. When the predicted results exceed 8 × 10^4^m³/d, further analysis incorporating other factors may be considered.

**Table 5:** Prediction performance for each category.

Category	Number of test set	precision	recall	F1-score
I	3	1	1	1
II	3	1	1	1
III	3	0.75	1	0.8571
IV	3	1	1	1
V	3	1	0.6667	0.8
VI	0			

## Results and discussion

By conducting a series of comparison experments, we ascertained that the IPHTSG prediction model can be effectively established utilizing a data-driven methodology grounded in engineering and production data. The creation of the IPHTSG database constituted the cornerstone for developing the IPHTSG prediction model, which served as a comprehensive sample database for model training purposes. The precision of this database is thus a critical determinant of the reliability of the IPHTSG prediction model. Consequently, during the database establishment process, meticulous data cleaning, standardization, and correlation analysis on the engineering and production data were imperative to ensure the high application value and integrity of the database^33–35^. Since missing data can lead to a decline in model performance and different machine learning algorithms have varying capabilities when handling missing data, if a dataset contains a large amount of missing data, it may be necessary to select more complex algorithms or perform additional data preprocessing, which increases both the difficulty of algorithm selection and computational costs. Noisy data can also cause model overfitting and reduce its generalizability. Therefore, before initiating model training, we cleaned the data by performing tasks such as correcting erroneous data, deleting duplicate data, and imputing missing data. Additionally, we conducted feature parameter selection through methods such as correlation analysis.

In this study, six widely applied machine learning algorithms were selected to construct the prediction model. Through a comprehensive comparison of the training set accuracy and multiple evaluation metrics for the test set, we found that only the RF, XGBoost, and CatBoost algorithms achieved accuracy rates exceeding 80% on each sample set, thus meeting the stringent requirements of the project. However, the model training time for the CatBoost algorithm is significantly longer than those of the other models, being at least 20 times greater than those of the RF and XGBoost models, which greatly reduces the training efficiency of the model. Compared with the RF model, the XGBoost model exhibits a similar prediction accuracy on the training set, both exceeding 99%. However, its test set performance in terms of the accuracy, precision, recall, and F1-score is 6.67% (93.33%), 3% (95%), 7.39% (94.12%), and 6.14% (93.14%) higher than that of the RF model, respectively. Therefore, for the case study developed in this research, the XGBoost algorithm was chosen to establish the IPHTSG prediction model.

Apart from the accuracy and reliability of the database and the intrinsic performance disparities among the algorithms, another significant factor contributing to the marked differences in the recognition accuracy among the various algorithms is the selection of the feature parameters. Due to certain constraints when obtaining actual field data within the scope of this study, only a subset of the engineering and production data was selected to provide the feature parameters. However, numerous logging data and other engineering parameters can also provide valuable insights into the characteristics of the IPHTSG. If circumstances permit, it is advisable to consider logging data and other engineering parameters concurrently, and to expand the feature parameters for model training to further enhance the model’s predictive performance. In the case study presented in this paper, two parameters, the OFC (the influence weight exceeds 0.4) and the ERL (the influence weight exceeds 0.2), significantly affect the prediction results. The analysis revealed that the ERL of horizontal wells is positively correlated with both the reservoir contact area and the number of seepage channels; as the ERL of horizontal wells increases, both the contact area with the reservoir, and the number of seepage channels increase as well. This phenomenon is more conducive to the flow of natural gas into the wellbore, thereby increasing the initial production rate. Moreover, when the ERL increases, the pressure gradient around the wellbore also changes. A reasonable distribution of the pressure gradient facilitates a more efficient inflow of natural gas into the wellbore, increasing the OFC and consequently boosting the initial production rate.

For the case study in this research, the optimized XGBoost prediction model has relatively poor prediction performance for the samples in the V category. Through analysis, we believe that there are two main reasons for this phenomenon. First, the sample size is insufficient, making it difficult to accurately capture the characteristics of some data. Second, the geological and engineering factors of tight sandstone reservoirs are relatively complex. For wells with higher initial production capacity, the reservoirs in which they are located may have better physical properties and greater degrees of fracture development. However, in this case study, data related to the degree of fracture development were not collected. As a result, comprehensively considering these complex geological factors is challenging. In future research, efforts should be made to collect a wider variety of parameters to achieve data comprehensiveness.

To achieve accurate prediction of the IPHTSG, it is imperative to increase the number of samples in the database and refine the classification step size for the initial production capacity, as a reduced step size facilitates more precise guidance for actual field development and production activities. On the basis of the case study presented, the method proposed in this paper can predict the IPHTSG to a certain extent, thereby offering a novel and innovative technical approach for its prediction and evaluation.

## Conclusions

To address the challenge of accurately predicting the IPHTSG, this paper introduces a new data-driven method based on partial engineering and production data, and the feasibility of this method is verified through the application of an actual case. Some meaningful conclusions are listed below.

- An IPHTSG database for the study area was established, and it ensures one-to-one correspondence between the feature parameters and the target category and provides a sample database for the establishment of the IPHTSG prediction model.
- An IPHTSG prediction model for the study area was established. By using five feature parameters, namely, the ERL, VT, OFC, BHP, and ASI, as sample data, we established an IPHTSG prediction model employing the OVR SVMs, OVO SVMs, RF, NN, XGBoost, and CatBoost algorithms. The parameters for each model were rigorously optimized through grid search and 10-fold cross-validation methods. On the basis of the recognition accuracy on the training sets and the accuracy, precision, recall, and F1 score evaluation metrics on the test set across the six target categories, the XGBoost prediction model was selected as the optimal model.
- In this work, prediction of the IPHTSG was achieved, and the proposed method demonstrated feasibility. This approach will aid engineers in terms of formulating more rational development plans based on multiple factors, including geology and engineering. These plans encompass a good location layout, completion methods, and fracturing designs, ultimately enhancing the overall development efficiency and economic benefits. However, the selection of model feature parameters and machine learning algorithms needs to be tailored according to the characteristics of the data in the study area.
- In the future, this research can be integrated with real-time interpretation of logging-while-drilling data, real-time updating of reservoir geological models, and maximization of horizontal well productivity, thereby further integrating geology and engineering.

## Supplementary Information

Below is the link to the electronic supplementary material.

Supplementary Material 1

## Data Availability

Data is provided within the manuscript or supplementary information files. The relevant data has been submitted through the system with the file name “train-test-Scientific Reports.xlsx”.

## Abbreviations

IPHTSG: initial production in horizontal wells targeting tight sandstone gas reservoirs
OVR SVMs: one-versus-rest support vector machines
OVO SVMs: one-versus-one support vector machines
RF: random forest
NN: neural networks
XGBoost: extreme gradient boosting
CatBoost: categorical boosting
C: penalty coefficient
$$\sigma$$: standard deviation
$\sigma$: the parameter in the kernel function, = 1/^2
n_estimators: number of decision trees
max_features: subset of feature sets selected randomly
max_depth: maximum depth of each decision tree
learning_rate: controls the step size of each iteration to update the weights
epochs: the number of times the entire training dataset
batch_size: the number of samples that are processed simultaneously
